# Modelling and simulation of smart city drainage system based on digital twin five-dimensional models

**DOI:** 10.1371/journal.pone.0352787

**Published:** 2026-07-09

**Authors:** Yuanjing Zhao, Min Yang

**Affiliations:** College of Civil Engineering, Chongqing Vocational Institute of Engineering, Chongqing, China; Izmir Katip Celebi University: Izmir Katip Celebi Universitesi, TÜRKIYE

## Abstract

The construction of smart cities has entered a new stage of industrial development in light of the industrial revolution. The aim is to create digital twin cities that integrate the dual systems of physical and digital bodies. The drainage system of a city forms the foundation and core of its digital twin, hence the need for a construction and simulation study proposed in this research. The study is based on the five-dimensional model of the digital twin. First, the research aims to develop a five-dimensional digital twin model that incorporates twin data and connections, building upon a three-dimensional model to improve its applicability. Second, the research looks to create a digital twin drainage system model using a lightweight framework, employing a dynamic scheduling algorithm and model-view-controller to facilitate intelligent scheduling of the drainage system and enable real-time data collection and transmission. The experimental outcomes indicate that in normal conditions, the overflow loss of the mathematical twin drainage system was 30m^3^/s, 34m^3^/s, and 25m^3^/s under the conventional fixed-priority scheduling algorithm and dynamic scheduling digital twin drainage system algorithm, respectively. Due to the ratio of the improved overflow loss to the pre improved overflow loss being called the improvement ratio, the improvement ratios generated by the mathematical twin drainage system in different situations were 48.67%, 48.1%, and 48.57%, respectively, which significantly enhanced the performance and durability of the urban drainage system. The model effectively transforms and enhances the current urban drainage system by increasing the efficiency of scheduling pumping station clusters. It also offers valuable reference for the implementation of digital twin concept and technology.

## 1. Introduction

Due to the progress in technology and data science, Digital Twin (DT) technology is increasingly being recognized [1]. In today’s digital age, digital twin technology, as a key support for smart city construction, is leading urban systems towards a new stage of intelligence. Specifically, in urban systems, digital twins achieve comprehensive monitoring, simulation, prediction, and optimization control of urban infrastructure and various public service systems through precise modeling and data-driven virtual mirroring, providing a new perspective and effective means for solving complex urban problems [2,3]. The urban Drainage System (DS) is an essential aspect of urban infrastructure, critical to upholding citizens’ everyday life and economic activities [4]. The physical makeup and operational status of a drainage system includes precipitation events, surface runoff, and the state of the relevant drainage apparatus and waterways [5]. Nonetheless, current drainage systems confront difficulties resulting from factors such as climate change, urbanization progress, and deteriorating infrastructure [6]. It is crucial to find a methodology that can effectively enhance the prediction and management of DS performance [7]. Consequently, this study proposes a Smart City Drainage System (SCDS) that is based on Digital Twin Five-Dimensional Model (DT-5D-M). This system introduces the DT technology to construct an integrated, data-driven DT model with five dimensions, comprising three-dimensional spatial data and a temporal urban DS model. The goal is to advance the development of smart cities (SCs) in order to improve public quality of life. The contribution of the research is to complete the drainage design of cities through the construction of digital twin models, reducing the overflow loss of pump station clusters and providing practical solutions for solving urban waterlogging problems.

At present, digital twin technology is expanding its application scope with unprecedented vitality and deeply penetrating into various key areas of smart cities. In the field of transportation, digital twins focus on building real-time digital maps of road networks and vehicle operating status, and using intelligent algorithms to optimize signal timing and plan travel routes to alleviate congestion and improve traffic efficiency. The digital twin of the energy system focuses on fine modeling of energy production, transmission, and consumption processes, and uses data analysis to achieve energy supply and demand balance, fault warning, and energy conservation and efficiency improvement. The digital twin of environmental systems is dedicated to the dynamic simulation of factors such as air quality, water pollution, and ecological changes, providing scientific basis for environmental regulation, pollution control, and ecological protection decisions. The application of the five dimensional digital twin model designed for research in drainage systems lies in integrating three-dimensional space, time dimensions, twin data and connections, and accurately depicting the physical characteristics, operating status, and interaction relationships of drainage pipelines, pump stations, and sewage treatment plants. By using dynamic scheduling algorithms and lightweight development frameworks, intelligent monitoring of the drainage process, real-time data collection and transmission, and efficient coordination and control of pump station clusters can be achieved to cope with complex problems such as waterlogging and overflow caused by rainfall.

The study comprises seven main sections. Firstly, the introduction outlines the role of information technology in daily life domains amidst the rapid development of the digital era, presented objectively without any subjective evaluations. The second part is the research objective. The third part provides a literature review, summarizing the implementation of DT models and SCDS across various fields, and reporting the current research findings by numerous scholars. Section IV presents a study of SCDS design, based on DT-5D-M, for developing a model of urban DS. This section contains two sub-sections, the first one examines the construction of five-dimensional DT body models that incorporate twin data and connectivity, and the second one explores DTDS modelling based on a Dynamic Scheduling Algorithm, using the Model-View-Controller (Spring Spring MVC Mybatis, SSM) lightweight framework. Section V carries out simulation experiments on the urban distributed system (DS) of DT-5D-M. The experiments analyse the traditional Fixed Priority Scheduling (FPS) algorithm, Multi-Level Dynamic Priority And Importance Scheduling (MDPIS), and the DTDS algorithms in various scenarios. The experiments focus on urban DS analysis. Part six presents an overview of the research methodology and findings. The seventh part is about the shortcomings of the research, mainly analyzing and elaborating on the model data dependency and scheduling algorithm defects, simulation experiment scenarios, and regional applicability.

## 2. Objectives

This article is based on the five dimensional model of digital twins, and focuses on the practical needs of operation and control of smart city drainage systems, as well as flood prevention and control. It conducts modeling and simulation research on smart city drainage systems. The main research objectives include: firstly, integrating twin data and associated interaction dimensions into traditional 3D models, constructing a digital twin five dimensional model suitable for urban drainage scenarios, and improving the applicability and mapping accuracy of the model; Secondly, relying on the SSM lightweight architecture, combined with dynamic scheduling algorithms, a digital twin drainage system model is built to achieve real-time collection, transmission, and intelligent scheduling of operational data for drainage networks and pump station clusters; The third is to compare the overflow loss and drainage efficiency indicators of traditional fixed priority scheduling algorithms and improved dynamic scheduling algorithms through simulation, and verify the optimization effect of the proposed model and algorithm; The fourth is to provide theoretical reference and engineering practice basis for the practical application of digital twin technology in smart city drainage engineering, the improvement of pump station cluster scheduling efficiency, and urban waterlogging control.

## 3. Related work

The implementation of Digital Technology (DT) offers an enriching urban living experience by utilizing physical models in real-world city construction. Integrating it with a multi-scale and multi-probability simulation process, numerous scholars have conducted extensive research on it. Tao F et al. systematically analyzed the current research status of DT modelling. Firstly, they performed a comprehensive and profound analysis of DT modelling concerning application areas, levels, and disciplines. Based on the theoretical system of DT modelling proposed by previous scholars, the current study on DT modelling has been categorized and analyzed. The findings are subsequently presented, along with recommendations for future research [8]. The main questions answered by Semeraro C et al. what is DT, where is it appropriate to use DT, what are the main challenges in implementing DT. It also provides an up-to-date image of the main DT components, studied in different application areas and related technologies, clearly tracing current research advances and technical challenges in conceptualizing and building DTs [9]. In order to define the digital twin of rainwater and cross-border water security projects in the literature, Brasil J A T et al. used a literature review to obtain existing conceptual challenges and the definition of digital twin as a framework. They determined how the mathematical models reported in the literature could improve the development of digital twin and evaluated the potential benefits related to the application of digital twin in the National Bureau of Statistics. The results show that digital twins have demonstrated effective effects in the management and planning of urban water supply systems [10]. Kang J L et al. developed a DT model that matches the actual conditions of the plant, which was applied to a 25 kw large-scale solid oxide fuel cell system in which the system is connected to an upstream reformer, a downstream burner and multiple heat exchanger. The results of the study showed that the model can effectively help the operator to determine the operation strategy to execute the process safely and stably through simulation [11]. Lumley D and other experts designed a digital twin model based on a future urban flow platform to improve the real-time sewage treatment performance and predictability in the face of storms in cities. Through the multifunctionality of this model, they gained a deeper understanding of watershed dynamics. This model is embedded with a predictive control module that can provide set values for watershed control. The results show that the model can promote real-time sewage treatment in cities [12].

Sustainable drainage systems (SDS) are gaining increasing attention, yet their implementation is still in its nascent stage within the drainage sector. Addressing urban drainage issues necessitates exploring new approaches, and scholars are devoting considerable research to the development of SDS construction methods. Pedersen A N et al. defined four possible classifications of “uncertain positions” in the comprehensive urban drainage model in order to better replicate the physical system of urban drainage using digital twins, and developed a structured framework for identifying and diagnosing various types of errors. The results show that this method can iteratively improve the comprehensive urban drainage model [13]. Experts such as Lu J have considered the design of urban drainage systems by considering redundant pipe networks and using graph theory and adaptive genetic algorithms for preliminary layout and design of urban drainage systems. In addition, this study increases elasticity by introducing additional waterway paths (loops)/redundancy. The results show that when rainfall exceeds the design standards, the total overflow of urban drainage systems with pipeline redundancy is reduced by 20–30%, which is significantly better than the pipeline network without pipeline redundancy [14]. Ferrans P et al. showed how a decision support system for sustainable urban DS can be constructed, selected and used to help decision makers solve problems. Environmental and social factors, DS training performance and selection criteria, rainfall stochasticity and future scenario impacts are identified. They conclude with recommendations for sustainable urban DS to better assist decision makers in dealing with drainage challenges that arise in urban centre [15]. Zhang N and other researchers used building information modeling technology to automate the design of drainage networks, dividing the drainage network into smaller components at the geometric boundaries of pipeline panels. In addition, the study also generated a material list for each pipeline panel to further optimize cutting. The results show that this method has achieved automation in the design of drainage systems under the background of panel construction [16]. Guptha G C et al. conducted a comprehensive evaluation of drainage diagnostics in Gurgaon, India, using high resolution remote sensing datasets, storm-water management models for detailed analysis. The results of the study showed that climate change alone poses a more serious threat than that posed by urbanisation and will severely hamper the drainage capacity of DS [17].

In brief, the research on DT and DS remains deficient in terms of model precision and real-time concerns. Addressing these inadequacies, the present study offers a meticulous scrutiny of SCDS design and puts forward an investigation into the creation of a 5D DT physical model that integrates twin data and connectivity. This study allows for a more thorough understanding and modelling of the city’s data system, presenting the intricacies of the real world from a multitude of perspectives and introducing diverse dimensions to the model construction. In addition, the knowledge gap being addressed in this article is the realization of more efficient and intelligent drainage management through a digital twin five dimensional model, which improves the work efficiency of pump station clusters and reduces overflow losses, and verifies the potential application of digital twin technology in smart city drainage.

## 4. Design of SCDS based on DT-5D-M

In today’s information society, science and technology are leading the development of future cities, in which “SC” has become an important research field [18]. In the intelligent process of cities, DS, as a key part of infrastructure, but often face complex problems, such as unstable flow due to climate change, old facilities can not meet the needs of urbanisation [19]. Based on this, the study proposes a DT-5D-M based SCDS design, which utilizes DT technology and the principle of five-dimensional modelling to build a more comprehensive intelligent design model for DS. In order to be able to provide new ideas and tools for the optimization and management of DS in SC construction, and to promote the intelligent and humane development of urban infrastructure.

### 4.1. Five-dimensional DT body model construction incorporating twin data and connectivity

In order to promote the progress of urban drainage work, a virtual entity of the real urban drainage system, namely a five dimensional digital twin model, was constructed. Through the five dimensional digital twin model, it is possible to achieve bidirectional mapping between the physical world urban drainage system and the virtual image. In the five dimensional digital twin model, the urban drainage system includes street drainage pipe and channel systems, sewage pump stations and pressure pipelines, inspection wells, and sewage treatment plants. The operation of this model is to collect data from physical entities (real urban drainage systems) through sensors and embedded systems, and transmit this data to virtual entities (mapping of real urban drainage systems) through IoT communication protocols. Afterwards, the virtual entity uses the collected data for simulation analysis, generates control instructions or decision support, and performs service decision-making, feedback adjustment, and continuous iteration.

The development of DT body is gradually realized in more fields, and the application object of its creation is the DT model. The Beihang DT team expands the 3D model by adding two dimensions, twin data and connectivity, and proposes the concept of DT-5D-M [20]. The expression of their model is shown in Equation (1).


$$\begin{array}{c} Model=\left\{PE,VE,Ss,DD,CN\right\} \end{array}$$ (1)

**Fig 1:**
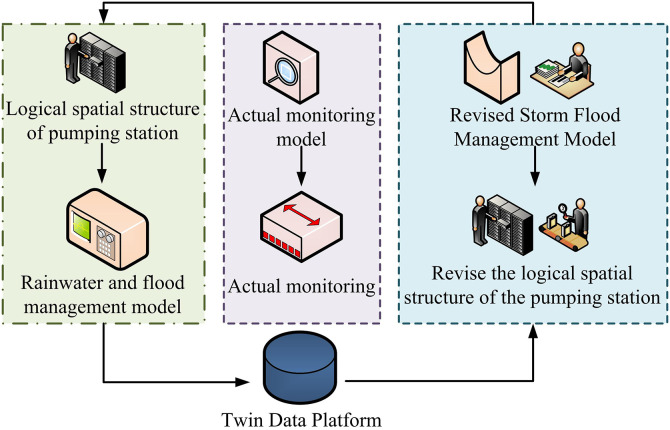
The process of establishing a digital twin model.

In Equation (1), PE denotes the physical entity, VE denotes the virtual entity, Ss denotes the service, DD denotes the twin data, and CN denotes the connection between the parts. The construction of the DT body of the SC needs to go through there are three stages: the extraction of the twin data, the construction of the twin model, and the combination and application of the twin model and data, in which the construction of the twin model is the core, so it is necessary to analyse the methodology of the construction of the twin model. The establishment of the DT model can be divided into three stages according to the time span: the design stage, the construction stage, and the feedback and correction stage. The establishment of the DT model process is shown in Fig 1.

During the design phase, the main components of the local DS need to be visited in the field and a management simulation model is built on the DS structure. In the construction phase, the location of equipment for collecting data, such as sensors and rain gauges, is selected based on the requirements of the model design [21]. In the feedback correction phase, the DT-5D-M is adjusted, as well as the priority parameters accordingly. It is especially important for constructing the DT-5D-M, which contains four components: virtual entities, services, twin data and connections. To construct DT-5D-M, physical entities (PEs) need to be maintained and analysed to obtain virtual entities (VEs). Therefore, the virtual entity is a mapping of the physical entity and describes the physical entity in terms of multi-dimensions and multi-scale, whose mathematical expression is shown in Equation (2).


$$\begin{array}{c} VE=\left\{G_{v},P_{v},B_{v},R_{v}\right\} \end{array}$$ (2)

**Fig 2:**
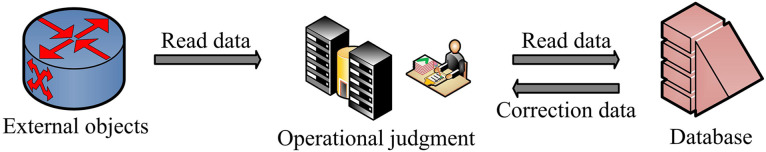
Workflow of digital twin service platform.

In Equation (2), $G_{v}$ denotes the 3D model describing the geometric parameters and relationships of the PE, $P_{v}$ denotes the addition of physical attributes and constraint-related information on the basis of $G_{v}$, $B_{v}$ denotes the corresponding behaviour of the PE in the context of the external environmental disturbances as well as the internal operational work mechanism, and $R_{v}$ denotes the law based on the associated data. While the service is the driving force of the DT system, the core is the computing platform that can realize the input and output functions to make decisions on the system operation scenarios and pumping station status in order to realize the scheduling of the pumping station clusters. Its workflow is shown in Fig 2.

The essence of the service is an arithmetic decision-making platform with input and output functions, and its specific processes are reading data, composing database, arithmetic decision-making, adjusting database, and standard output [22]. And the twin data are the data in the DT system that operate according to the rules of the system, which are used to study the operation of the DT system, as well as the flow and processing of data. The expression of its main components is shown in Equation (3).


$$\begin{array}{c} DD=\left\{D_{p},D_{v},D_{s},D_{k},D_{f}\right\} \end{array}$$ (3)

In Equation (3), $D_{p}$ denotes the physical attributes of the PE and the dynamic data reflecting the PE; $D_{v}$ denotes the geometric model data and the behavioral rule model data of the VE; $D_{s}$ denotes the data related to the required data processing methods and the analysis and training data in the operation process; and $D_{k}$ denotes the data related to the commonly used algorithm libraries and model libraries; $D_{f}$ denotes derived data obtained by means of pre-processing, integration, and fusion of $D_{p}$, $D_{v}$, $D_{s}$, and $D_{k}$. The service elements need to be driven by such system rules to operate the DT system. And the connection is to complete the connectivity between each component part, which connects the PE and VE structure points through the connection in order to form the topology of DT. The connection relationship between the components in the DT model is shown in Equation (4).


$$\begin{array}{c} CN=\left\{CN_{-}PD,CN_{-}PV,CN_{-}PS,CN_{-}VD,CN_{-}VS,CN_{-}SD,\right\} \end{array}$$ (4)

**Fig 3:**
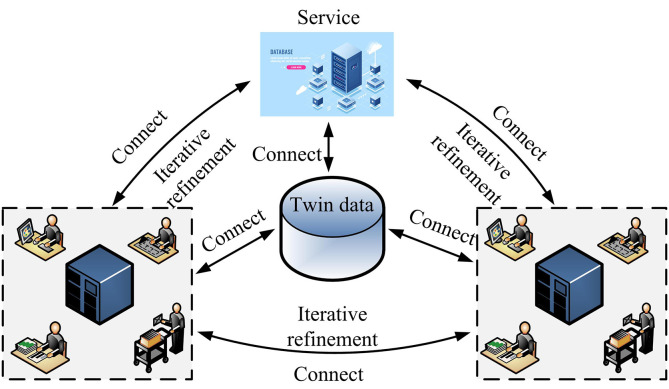
Five dimensional digital twin model.

In Equation (4), $CN_{-}PD$ denotes the interaction between PE and DD, $CN_{-}PV$ denotes the interaction between PE and VE, the collected PE data is transmitted, and the VE converts the results of the simulation analysis into control commands. $CN_{-}PS$ denotes the interaction between PE and service, the collected PE data arrives at the system platform through transmission, and the results of the service analysis are presented to the user through the mobile terminal. $CN_{-}VD$ denotes the interaction between the VE and the twin data, where the data generated by the VE is stored in the database through the database interface, and thus analyzed as historical data. $CN_{-}VS$ denotes the interaction between the VE and the service, where the data obtained from the model simulation is transferred to the platform through the network communication interface, and scheduling decisions are made through the algorithm. The $CN_{-}SD$ represents the interaction between the service and the twin data, and the storage of the data is done through a database interface or a persistence framework to get data that can be used as historical data or rule data for training and optimization of the data. The DT construction of the urban DS is performed according to the five dimensions of DT-5D-M. The main body of the construction mainly contains five parts: identifying physical entities, building virtual entities, designing data, establishing connections and realising services. It mainly focuses on the practical problems, improves the traditional scheduling process, and builds the platform for presenting data.DT-5D-M is shown in Fig 3.

**Fig 4:**
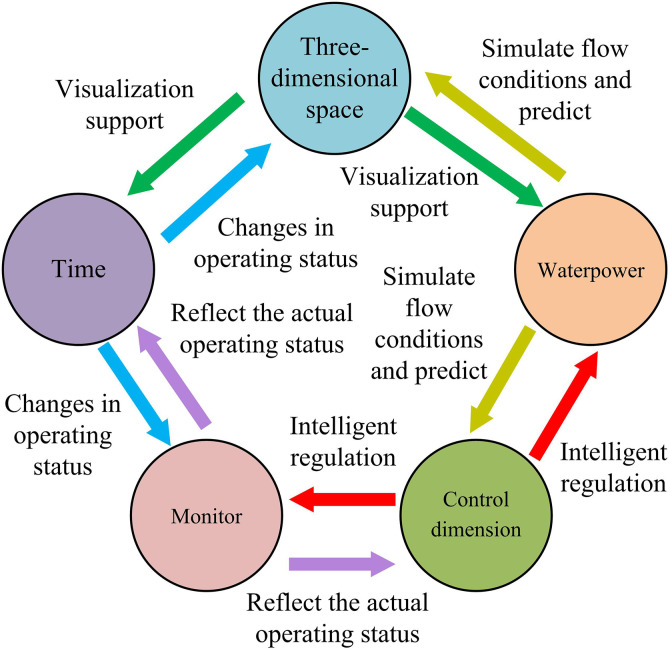
Schematic diagram of the interaction between different dimensions.

The system shown in Fig 3 requires real-time collection and transmission of data from the drainage scheduling process, including surface runoff, pipeline flow, cistern flow, and other critical data from various components. When the need for accurate measurement of flow data, the need to choose a volumetric flow meter to monitor the DT system to achieve real-time detection of various types of important flow information. In order to calculate the head of the pumping station in real time and obtain real-time processing flow and water pressure conditions, the need to use pressure instrumentation, can help workers accurately regulate the pumping station’s discharge, to prevent undue pressure and damage to the pipeline. In addition to the need to use level instrumentation for real-time monitoring of the pumping station reservoir level changes, in order to be able to quickly and accurately determine whether the pumping station has entered the emergency state, so as to take appropriate rescue measures [23]. The schematic diagram of the interaction between different dimensions is shown in Fig 4.

From Fig 4, it can be seen that the five dimensional modeling includes dimensions of three-dimensional space, time, hydraulics, monitoring, and control. Among them, the three-dimensional spatial dimension presents the precise location and geometric shape of drainage system facilities, providing visual support for planning and management. The time dimension covers the real-time dynamic process of system operation and can reflect changes in operating status during different time periods. The hydraulic dimension focuses on the laws and characteristics of water flow movement, simulates flow conditions, and predicts problems. In addition, the monitoring dimension obtains real-time data through sensors, reflecting the actual operating status and supporting calibration verification. The control dimension is intelligently regulated based on monitoring data and model predictions, achieving automated optimization scheduling. All dimensions are interrelated and collaborate to form an organic whole, achieving comprehensive digital mapping and intelligent management of urban drainage systems.

### 4.2. DTDS modelling based on dynamic scheduling algorithm with SSM lightweight framework

**Fig 5:**
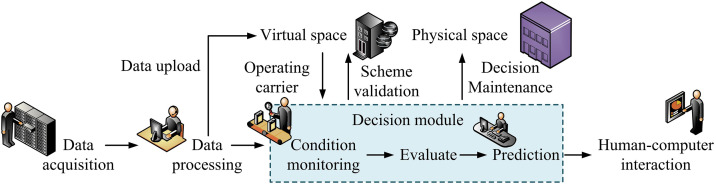
Intelligent decision-making process based on digital twins.

During periods of rainfall, DS leads to overflow losses in two primary ways. The first is urban flooding resulting from insufficient capacity of pumping stations, while the second is water pollution due to the direct discharge of untreated mixed sewage [24]. Meanwhile, the System State (SS) of the DS includes emergency and safety states. This may be calibrated based on the overall performance of the DS, weather conditions, system pressure, etc. Schedule Cycle (ScC) is the cycle of adjusting the pumping station’s discharge accomplished by the controller based on the scheduling decisions, while the development and implementation of scheduling strategies are the core aspects of DS management [25]. Scheduling decision-making is to dynamically adjust the pumping station discharge according to the state of the pumping station cluster to achieve the purpose of avoiding overflow, as well as reducing the overflow loss. Combining intelligent decision making with DT technology, its structure is shown in Fig 5.

**Fig 6:**
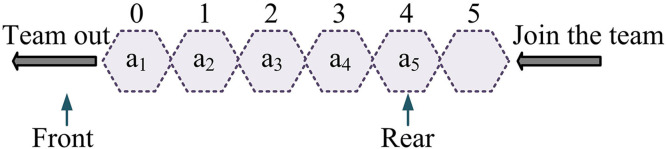
The process of joining the pump station nodes.

The scheduling decision is broken down into two stages. The first stage involves choosing the pumping station with the best priority, which is initialized by the scheduling rules (SR) in a secure condition, and then adjusting the emissions in accordance with the scheduling principles. The second level is to restore the emergency pumping station to the normal state, firstly, the current urgency value of the emergency pumping station is sorted. And then the emergency pumping station node with high emergency value is queued, and the emergency pumping station nodes are traversed according to the order of the first-in-first-out queue. The process schematic is shown in Fig 6.

**Fig 7:**
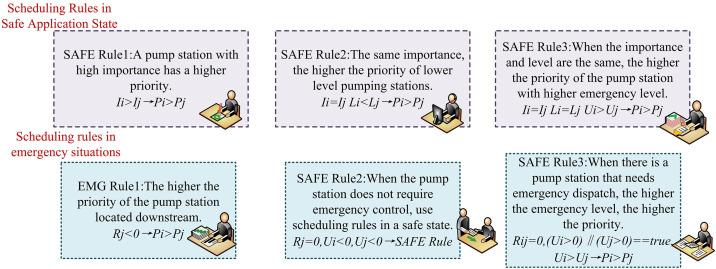
SR in safe and emergency states.

The SR of the pumping stations are based on the different states and situations of the pumping stations to determine the priority of the pumping stations [26]. The purpose of the pumping system is to conduct centralized dispatching of urban drainage to achieve the effect of saving manpower and safe and high-quality water supply. The system is mainly placed in the drainage self-control system of the digital twin drainage system. In addition, the system is a real-world system that can be applied in reality. In this study, there are two different types of SR. First, while the pumping station cluster is running, the SR initialises the priority of each pumping station in the safe condition to decide the pumping station’s initial discharge. The second is when the pump station cluster is in an emergency state, the SR adjusts the priority of the pump stations and adjusts the discharge of the emergency pump station and the upstream pump station cluster, so that the emergency pump station returns to the safe state. The steps of the SR for pumping stations are shown in Fig 7.

Based on scheduling choices made during the operation of the urban DS process, the overflow loss of the pumping station group is reduced, increasing drainage effectiveness. The overflow loss occurs in the equation indicated in Equation (5) when the present water level $H_{c}$ in the catchment well volume pool of the pumping station reaches the maximum water level line $H_{max}$, as suggested by the real-life scenario used to compute the overflow loss of the DS pumping station.


$$\begin{array}{c} \Delta Q=I\times (Q_{out}-Q_{in}) \end{array}$$ (5)

In Equation (5), $Q_{in}$ indicates the inlet flow rate of the pumping station, $Q_{out}$ indicates the treatment flow rate of the pumping station, and I indicates the water velocity of the flowing water in the drainage pipe. In the hydraulic calculation of the drainage pipe is widely used in the open channel uniform flow equation, the basic equation expression shown in Equation (6).


$$\begin{array}{c} Q_{in}=Av \end{array}$$ (6)

In Equation (6), A represents the area of the over-water section, which is surrounded by the water surface line and the bottom surface of the pipe at a certain point in time, and v represents the over-water flow rate. For the calculation of the flow rate, Xie Cai’s equation was used and its mathematical expression is shown in Equation (7).


$$\begin{array}{c} v=C\sqrt{RJ} \end{array}$$ (7)

In Equation (7), R denotes the hydraulic radius, which is the ratio of the cross sectional area of the over-water to the wet week at a certain point in time, J denotes the hydraulic gradient, and C denotes the Xie Cai coefficient. The Xie Cai coefficient is typically utilized in Manning’s equation. The mathematical expression is shown in Equation (8).


$$\begin{array}{c} C=\frac{R^{\frac{\text{1}}{\text{6}}}}{n} \end{array}$$ (8)

In Equation (8), n represents the pipe wall roughness coefficient. Therefore, the equation for calculating the flow rate of the inlet pipe of the pumping station is shown in Equation (9).


$$\begin{array}{c} Q_{in}=A\times \frac{R^{\frac{\text{2}}{\text{3}}}\times J^{\frac{\text{1}}{\text{2}}}}{n} \end{array}$$ (9)

In reality, however, the head depends on the pump’s working capacity, and Equation (10) illustrates the connection between the head and the treated flow rate.


$$\begin{array}{c} H=\frac{\overset{\text{2}}{\underset{\text{2}}{R}}\omega -\frac{QR_{\text{2}}\omega cos\beta_{\text{2}}}{2\pi R_{\text{2}}b_{\text{2}}}}{g} \end{array}$$ (10)

In Equation (10), $R_{\text{2}}$ for the distance between the vane outlet and the pump shaft’s centre, H stands for the actual head, ω A for the angular velocity of vane rotation, Q for the actual flow rate, $\beta_{\text{2}}$ for the outlet mounting angle, $b_{\text{2}}$ for the size of the vane outlet, and g for the unit gravitational acceleration. Equation (11) illustrates how to use the head to compute the handling flow rate of the pumping station.


$$\begin{array}{c} Q_{out}=\sum_{i=1}^{n}F\left(h\right)\times p\left(i\right) \end{array}$$ (11)

F(h) represents the fitted pump head and flow rate curve in Equation (11), and p(i) represents the condition of the i th pump. Equation (12) illustrates the equation for the pumping station overflow loss.


$$\begin{array}{c} loss=\sum_{i=0}^{n}(I_{i}\times \Delta Q_{i})=\sum_{i=0}^{n}(I_{i}\times \left\{\sum_{j=1}^{n}\left[F\left(h\right)\times p\left(j\right)\right]-A_{i}\frac{R^{\frac{\text{2}}{\text{3}}}\times J^{\frac{\text{1}}{\text{2}}}}{n}\right\}) \end{array}$$ (12)

**Fig 8:**
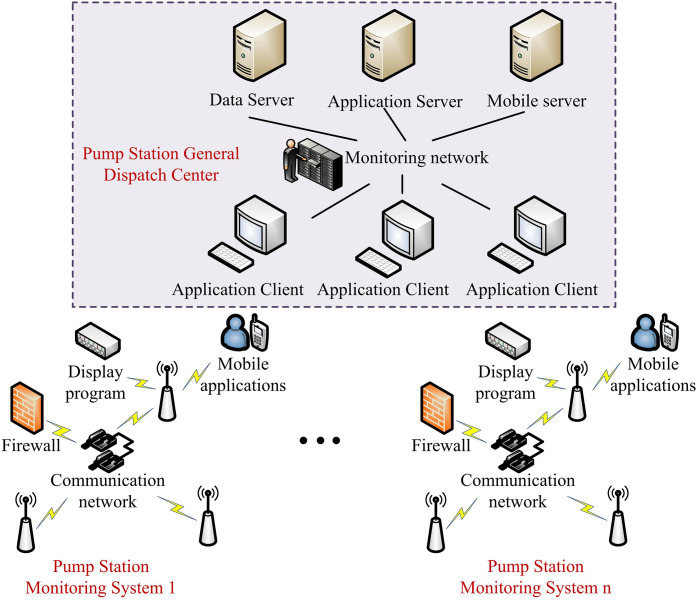
Deployment structure of drainage automatic control system.

In Equation (12), i, j indicates that the overflow loss occurring in the pump house is calculated in turn, and the DT model is dispatched in accordance with the principle of giving up the high and low importance in the pump station cluster, so that the overflow loss can be minimized. By calculating the overflow loss of the pumping station cluster, while centralized scheduling, real-time management can be achieved, to a certain extent, to achieve the effect of safe and high-quality water supply. The automatic control system for urban drainage is shown in Fig 8.

The scheduling state of the pumping station under the automatic control system of this urban drainage is determined, firstly, whether the pumping station can be operated properly, and secondly, the extent to which the pumping station is able to complete the drainage work. In this research design DS, the state of the pumping station is divided into three scheduling states. The first one is the fully dispatch-able state, when the maximum discharge of the pumping station and the residual discharge capacity of the corresponding pumping station can meet the required pumping capacity of the pumping station, and its mathematical expression is shown in Equation (13).


$$\begin{array}{c} (Q_{in}<Q_{out})+(Q_{in}<Q_{surplus})=ture\to Scheduled \end{array}$$ (13)

The second is the dispatchable state, when the maximum discharge of the pumping station or the residual discharge capacity of the corresponding pumping station cannot satisfy the required pumping capacity of the pumping station, the mathematical expression of which is shown in Equation (14).


$$\begin{array}{c} \left\{ \begin{array}{c} @c@\;A=0,\;B=0\\ Q_{in}<Q_{out}\to A=1,Q_{in}<Q_{surplus}\to B=1\\ \;A\wedge B=1\to Partially\;Scheduled \end{array}\right. \end{array}$$ (14)

Furthermore, it is a non-dispatchable state, when the maximum discharge of the pumping station and the residual discharge capacity of the corresponding pumping station cannot satisfy the required pumping capacity of the pumping station, the mathematical expression of which is shown in Equation (15).


$$\begin{array}{c} \left[(Q_{in}<Q_{out})\|(Q_{in}<Q_{surplus})\right]=ture\to UnScheduled \end{array}$$ (15)

Based on the aforementioned three different pumping station states, it was determined that the DTDS designed for this study will calculate the head in real time by pressure vacuum gauge. In order to identify visually whether the pumping station is in an emergency status or not, the level metre is utilised to acquire the real-time level change of the pumping station reservoir flow [27].

## 5. Urban DS simulation experiment based on DT-5D-M

In order to provide a clearer picture of the impact on system operation, a more comprehensive understanding of the different physical parameters as well as the pipeline parameters is required. The DT data model needs to be tested and validated more comprehensively in terms of initial data and experimental parameters. In the framework of DT data, the initial data is labelled as the Dp part, indicating a direct correlation with the physical entities. In the DT data system, the experimental parameters provide detailed settings for the experimental process, providing a predetermined environment and imposed conditions for the experiment to ensure that more efficient and accurate results can be obtained from it. In the Ds part of the DT model, the parameters include the set parameters of the experimental group and the parameters of the catchment volume, and the settings of the experimental environment include the environmental parameters such as temperature, humidity, and air pressure, which may have an impact on the performance, efficiency, and stability of the DS. Meanwhile, the setting of the experimental environment also involves the maintenance and protection of the experimental equipment to ensure the stable operation of the experimental equipment in the harsh environment.

### 5.1. Results of mathematical twin DS operation in different scenarios

The validation process of a model is an important step in ensuring its accuracy and reliability. The study comprehensively validated the model using historical data. The study collected operational data of the drainage system over the past 5 years (2020–2024), including rainfall events, pump station operation status, pipeline flow rate, and water level changes. These data come from the city’s drainage monitoring network, covering rainfall events of different seasons and intensities, as well as corresponding system responses. In addition, rainfall data mainly comes from corresponding meteorological departments, and geographic information data is mainly collected through geographic information system technology, such as terrain elevation, land use type, road network, etc. After collecting the data, the study preprocessed it, removed outliers and missing values, and standardized the data to ensure consistency and comparability. In addition, the study calibrated the model parameters using some historical data and adjusted key parameters in the model, such as pipeline roughness and pump station efficiency, to make the simulation results of the model closer to the actual observed values. Afterwards, the simulation results of the model will be compared with the actual drainage situation in historical data to evaluate the accuracy of the model. By the way, The verification methods for real drainage networks mainly include field monitoring data comparison method, field investigation and empirical verification method, historical event retrospective verification method, and model mutual comparison method, and the accuracy of the model can be quantified through field monitoring data comparison.

In terms of evaluation indicators, the study selected Root Mean Square Error (RMSE) and coefficient of determination R^2^. RMSE is used to measure the difference between simulated results and actual observations, while R^2^ is used to measure the correlation between model predictions and actual observations. The comparison of the validation results of the model under different rainfall events is shown in Table 1.

**Table 1:** Comparison of validation results of the model under different rainfall events.

Types of rainfall events	Actual drainage efficiency/%	Model predicted drainage efficiency/%	Error/%	RMSE	R^2^
Light rain	85.5	83.6	1.9	0.56	0.92
Moderate rain	78.4	76.9	1.5	0.72	0.89
Rainstorm	62.7	60.3	2.4	1.23	0.85

From Table 1, it can be seen that in light rain and moderate rain events, the errors between the predicted drainage efficiency of the model and the actual value are 1.9% and 1.5%, respectively, while the RMSE is 0.56 and 0.72, and the R² is 0.92 and 0.89, respectively. This indicates that the model has high accuracy and reliability in simulating drainage efficiency. In the rainstorm event, the error is 2.4%, RMSE is 1.23, and R ² is 0.85. The model can still simulate the change trend of the drainage system well. In summary, the model exhibits good performance under different rainfall conditions, which can provide strong support for the optimization and management of drainage systems.

In addition, the detailed boundary condition specifications for hydraulic modeling include flow boundary, water level boundary, hydraulic slope boundary, and time boundary. Among them, the flow boundary is set based on the actual rainwater collection and upstream water situation to determine the inlet flow, and the outlet is determined based on the receiving water body or downstream pipe network situation. The water level boundary focuses on the actual elevation and design water level range of key nodes, while the hydraulic slope boundary needs to be set according to the pipeline laying slope and terrain. Meanwhile, the time boundary takes into account the duration of rainfall events and changes in rainfall intensity.

**Fig 9:**
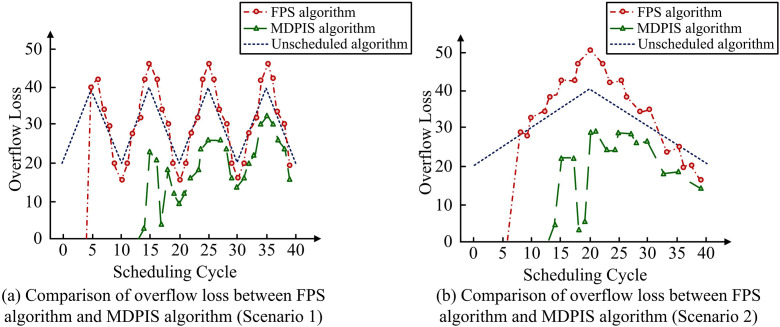
Change curve of overflow loss generated by scenarios 1 and 2 under FPS and MDPIS algorithm scheduling.

Under conditions of various catchment parameters, the average overflow losses demonstrated by the FPS method and the MDPIS algorithm were investigated in depth. Specific numerical simulations were carried out to measure the associated average overflow losses, as well as a loss analysis of the MDPIS algorithm’s performance under different catchment parameters. Secondly, the overflow losses under normal conditions were assessed and the losses incurred when the optimal scheduling algorithm was not used were evaluated. The overflow losses caused under FPS algorithm, normal case and MDPIS algorithm and their trends. The trends are shown in Fig 9.

**Fig 10:**
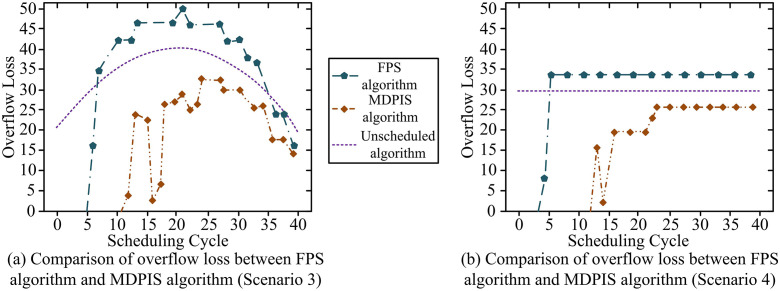
Change curve of overflow loss generated by scenarios 3 and 4 under FPS and MDPIS algorithm scheduling.

Fig 9 shows that the stability to time jitter is better and the difference in overflow loss is larger when different scheduling algorithms are used. The example using the FPS algorithm has more overflow losses and a more severe overflow than the traditional case. Whereas the case of scheduling using MDPIS algorithm shows good results and significantly reduces the overflow loss. In Fig 9(a), the overflow loss of the FPS algorithm occurs at the 4th ScC with a high overflow loss value of 41, and the loss value is maintained at a relatively high level in the subsequent cycles. After the improved MDPIS algorithm, the overflow loss occurs only at the 13th ScC with a relatively low loss value of 21 and the highest loss value does not exceed 30. In Fig 9(b), the FPS algorithm experiences an overflow loss at the 7th ScC with a loss value of 28 and reaches the highest loss value of 52 in the follow-up. In contrast, compared to the FPS algorithm, the improved MDPIS algorithm occurs an overflow loss only at the 13th ScC with a loss value of 23, and the highest loss value is only 28. It can be seen that, in practical DS scheduling, the MDPIS algorithm can reduce the overflow loss more efficiently compared to the FPS algorithm, prolongs the time of occurrence of the overflow loss, and maintains the value of the overflow loss at a lower level. The trend of overflow loss in different scenarios is shown in Fig 10.

In Fig 10, the ability to limit overflow losses by scheduling with the MDPIS method remains at the original level, while the FPS approach causes more overflow losses than the ordinary situation. In Fig 10(a), the FPS algorithm for scheduling causes overflow loss at the 6th ScC with a maximum loss value of 51, while the improved MDPIS algorithm is the one that causes overflow loss at the 12th ScC with a loss value of only 4 and a maximum loss value of 34. In Fig 10(b), the FPS algorithm for scheduling occurs overflow loss at the 6th ScC with a loss value of 34, and the loss situation is stable thereafter. While the improved MDPIS algorithm is overflow loss occurs at the 12th ScC and its loss value is 16 and the highest loss value is also 26 and the overflow loss situation is steady state. The results of the overflow loss water volume caused by the four catchment parameter scenarios using the FPS algorithm for scheduling the pumping station clusters before improvement and the MDPIS algorithm for scheduling the pumping station clusters after improvement are shown in Table 2.

**Table 2:** Pump station overflow meter.

Sink group	Group1		Group2		Group3	
Experiential group	Before improvement	After improvement	Before improvement	After improvement	Before improvement	After improvement
Group 1	1112	581	1120	594	1341	716
Group 2	1108	550	1123	568	1342	704
Group 3	1103	571	1121	584	1341	701
Group 4	1105	537	1120	556	1341	686
Group 5	1107	576	1122	594	1340	712
Group 6	1110	572	1120	584	1341	703
Group 7	1098	563	1119	559	1335	679
Group 8	1099	512	1117	530	1331	657
Group 9	1103	550	1118	561	1332	674

In Table 2, there is a significant difference in the overflow performance of different drainage groups before and after the improvement. Each experimental group had significantly lower overflow figures after the improvements than before the improvements. Group 1 had an overflow value of 1112 before the improvements, which was reduced to 581 after the improvements. The same trend was observed in the other experimental groups and drainage clusters. This shows that the upgrades greatly decreased the overflow loss figures and raised the pumping station’s overall efficiency. The ratio of the overflow loss following the improvement to the overflow loss before to the improvement is known as the improvement ratio, and the results are displayed in Table 3 in order to more clearly demonstrate the decreased overflow loss of the improved MDPIS algorithm.

**Table 3:** MDPIS algorithm improvement comparison table.

Improvement ratio		Group 1	Group 2	Group 3	Average improvement ratio
Experimental group	Group 1	48.27%	47.39%	48.29%	47.97%
	Group 2	50.21%	49.64%	48.16%	49.19%
	Group 3	48.32%	48.15%	47.79%	48.09%
	Group 4	48.13%	50.87%	49.27%	49.42%
	Group 5	48.16%	47.19%	46.94%	47.43%
	Group 6	48.33%	48.21%	47.38%	47.98%
	Group 7	50.24%	50.21%	49.32%	49.93%
Average improvement ratio		41.98%	48.67%	48.17%	48.57%

Table 3 shows that the MDPIS algorithm shows significant superiority over the FPS algorithm when faced with fluctuating rainfall. The algorithm achieves the highest average improvement ratio of 49.81%, and in general, its improvement ratio fluctuates up and down within a range of about 48%. By observing the operational results, it is clearly observed that there is a significant correlation between the overflow loss and catchment parameters. It is also worth noting that the optimized MDPIS algorithm is capable of dynamically adjusting the priority levels, which will help to fully utilize the storage and discharge capacity of the pumping station. The different values of parameters will have an impact on the performance of the model. Taking parameters such as pipeline roughness coefficient, pump station efficiency, and rainwater runoff coefficient as examples, their sensitivity analysis is shown in Table 4.

**Table 4:** Sensitivity analysis of key parameters.

Parameter	Value	Drainage efficiency	Accumulated flood area	Parameter	Value	Drainage efficiency	Accumulated flood area
Efficiency of pumping station	0.60	+4%	−3%	Pipeline roughness coefficient	0.012	+8%	+6%
	0.65	+8%	−6%		0.014	Normal	Normal
	0.70	+12%	−9%		0.016	−10%	+8%
Rainwater runoff coefficient	0.45	−6%	+4%		0.018	−13%	+11%
	0.50	−12%	+9%	\	\	\	\
	0.55	−18%	+15%	\	\	\	\

From Table 4, it can be seen that different values of pump station efficiency, rainwater runoff coefficient, and pipeline roughness coefficient have a significant impact on the performance of the drainage system. Taking pump station efficiency as an example, when it increases from 0.60 to 0.70, the drainage efficiency increases from+4% to+12%. This indicates that improving pump station efficiency can significantly enhance the effectiveness of the drainage system and reduce the risk of urban waterlogging. When the rainwater runoff coefficient is 0.45, the drainage efficiency decreases by 6% and the accumulated water area increases by 4%. When the coefficient rises to 0.55, the drainage efficiency drops significantly to 18% and the accumulated water area increases to 15%. This indicates that an increase in the runoff coefficient of rainwater will put greater drainage pressure on the drainage system, and the accumulated water area will also significantly increase, reflecting the enormous challenges that urban drainage systems may face during rainfall. In addition, when the roughness coefficient of the pipeline is 0.012, the drainage efficiency increases by 8%, but when it increases to 0.018, the drainage efficiency decreases by 13%. This indicates that an increase in the roughness coefficient of the pipeline will lead to an increase in water flow resistance, a decrease in drainage efficiency, and further affect the change of accumulated water area, which has a negative impact on the performance of the drainage system.

### 5.2. Analysis of urban DS experiments with DT-5D-M

According to the actual DS design requirements of the pump is best to choose the same model, the number of units between 2 to 8. When a large number of precipitation pumping station needs to be configured with up to two different flow rate pumps, the pumping station is usually selected single-frequency pumps work in parallel, so this paper is mainly aimed at the pumping unit consisting of three single-frequency pumps optimization study. The pool and pump parameters are set as shown in Table 5.

**Table 5:** Design parameters of pool and pump.

Design parameters of water tank		Pump design parameter	
Design parameters	Numerical value	Design parameter	Numerical value
Effective volume/m^3^	2400	Number of pumps/Taiwan	3
Pool floor area/m^2^	500	Total design flow/m^3^·s^-1^	4.8
Pool floor elevation/m	30	Pump flow/m^3^·s^-1^	1.6
Design maximum water level/m	5.8	Pump flow/m^3^·s^-1^	1.6
Design minimum water level/m	1	Working mode	Parallel

**Fig 11:**
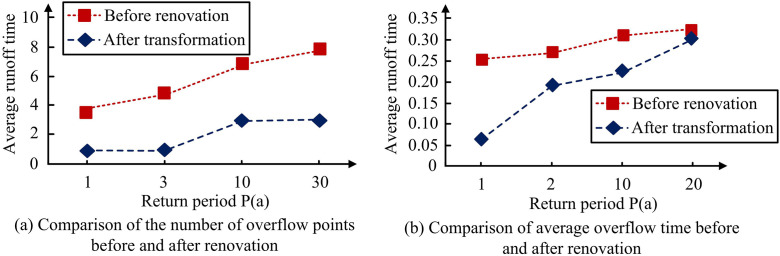
Comparison curve of the number of overflow points and overflow time of urban drainage systems before and after renovation.

In setting each pump to start pumping water level should be in accordance with the low to high reasonable settings, so that when the drainage is small only a pump work, with the increase in drainage, and then turn on the other pumps in turn. Set up a number of pumps to take turns to start the sequence, in which the starting level of each pump for the next pump to stop the water level. Set the pump start level to simulate the process of starting and stopping pumps. Facing the DS parameter settings, the experimental analysis of the overflow loss is as follows, in which the number of overflow points and the average overflow time of the change curve is shown in Fig 11.

**Fig 12:**
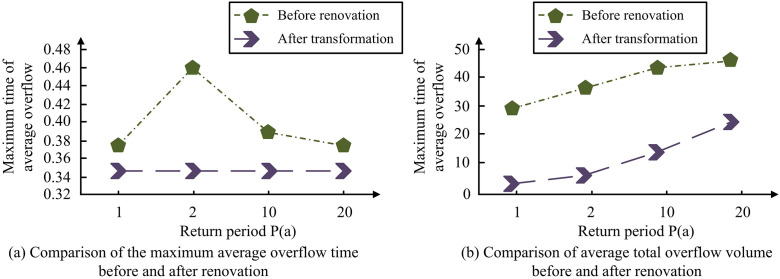
Comparison curve of average overflow flow and average overflow volume of urban drainage system before and after renovation.

In Fig 11, with the variation of the reproduction period, the observed number of overflow points and the average overflow time show a clear increasing trend, reflecting the corresponding changes before and after the improvement. In Fig 11(a), the difference between the number of overflow points before and after the improvement is 4 at 1P(a) during the variation process; while at 20P(a), this difference becomes 6. In Fig 11(b), the average overflow time similarly exhibits an increasing trend with the variation of the reproduction period. In particular, at 1P(a), the difference between the overflow time before and after the improvement is 0.20; however, at 20P(a), this difference narrows to 0.01. And the changes in the average overflow maximum time and the average total overflow volume are shown in Fig 12.

**Fig 13:**
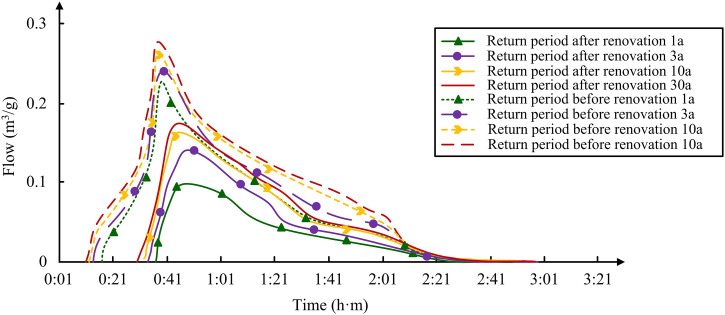
Comparison of discharge rates of urban drainage systems before and after renovation.

In Fig 12, with the change in the reproduction period, there is a more pronounced effect on the change in the maximum time of the average overflow volume and the average total overflow volume before and after the modification of the algorithm, which increased both before and after the modification. In Fig 12(a), the difference between the maximum time of mean overflow before and after modification is 0.03 at 1P(a), and remains 0.03 at 20P(a), but varies dramatically over the test interval, with a mean overflow before modification of 0.46 and a maximum of 0.34 after modification at 2P(a). In Fig 12(b), the mean time of mean overflow before and after modification also shows an increase in the time of overflow with the reproducible period shows an increasing trend, with the difference being 27 at 1P(a) and narrowing to 23 at 20P(a). The flow at the study area discharge before and after the urban drainage project under the four rainfall recurrence periods of P = 1a, P = 3a, P = 10a, and P = 20a is shown in Fig 13.

As can be seen in Fig 13, the trend of peak outfall flows being able to be reduced and delayed in occurrence at lower storm-water return periods, with a high percentage reduction in peak outfall flows, the City has a more pronounced effect on controlling peak outfall flows for storm-water at lower return periods. The occurrence of peak flows was delayed by more than 9 minutes for the 1-year and 3-year return periods, and this delay was 8 minutes. This shows that the ability of the urban DS to delay the time of occurrence of peak flow at the outfall is more prominent in response to the small return period rainfall scenario than in response to the large return period rainfall of more than 10 years.

## 6. Conclusion

With the advancement of information technology, data and information demonstrate explosive exponential growth, resulting in significant life changes for individuals and unprecedented development in the construction and management of cities. Therefore, smart cities have become a new trend of development. However, most researchers have not achieved information interoperability and synchronization between physical entities and digital bodies when digitising buildings. Therefore, this paper proposes a modelling and simulation study using SCDS under DT-5D-M, alongside experimental validation of the proposed system and algorithm. The findings demonstrate a noticeable increase in both the observed number of overflow points and the average overflow time with variations in the reproduction period. Specifically, at 1P(a), there was a difference of 4 overflow points before and after the improvement, and 6 at 20P(a). Additionally, the average overflow time showed an increasing trend as the reproduction period varied. At 1P(a), there was a 0.20 difference in overflow time before and after the algorithm was modified. However, at 20P(a), this difference narrowed to 0.01. The change in reproduction period had a more pronounced effect on the maximum time of the average overflow volume and the average total overflow volume before and after the modification. These values increased both before and after the modification. The maximum time of mean overflow demonstrated a difference of 0.03 at 1P(a) before and after modification, which remained 0.03 at 20P(a). However, it varied significantly over the period, where the mean overflow of 0.46 was observed before modification and a peak overflow of 0.34 after modification occurred at 2P(a). Additionally, the mean time of mean overflow before and after modification tended to increase during the reproduction period. The difference between 1P(a) was 27 which narrowed down to 23 at 20P(a). The mean overflow time before and after modification showed an upward trend throughout the reproduction period. The difference was 0.03 at 1P(a) and decreased to 23 at 2P(a), ultimately narrowing to 23 at 20P(a). The difference was 0.03 at 1P(a) and decreased to 23 at 2P(a), ultimately narrowing to 23 at 20P(a). The study offers solutions and suggestions for the development of digital twin supply chain (DTSC), serving as a reference for the practical application of DT. However, the model proposed in this research has a strong dependency on the initial model and fails to ensure the complete exchange of information between physical and virtual entities. Therefore, additional research is necessary for the effective implementation of DT technology in supply chain management.

The research and design of digital twin models have strong generalization and scalability. It can adapt to the layout and component differences of different urban drainage systems. By adjusting model parameters or modifying rule sets and parameter constraints, it can meet different drainage standards and regulatory requirements in various regions. It can also dynamically adjust data input and operation modes according to the richness of data. In addition, the model can also add new functional modules according to actual needs, such as simulating green infrastructure and integrating new data sources such as water quality and water temperature sensor data. However, in areas with poor data quality or severe missing data, the model may require more data preprocessing and supplementary work, and its generalization and scalability need to be continuously validated and optimized in practical applications.

To achieve the application of smart city drainage system simulation based on digital twin five dimensional model in the real world, it is necessary to build a complete system that tightly integrates software and hardware. On the software side, develop an intelligent platform that integrates data collection, simulation, decision-making, and visualization functions. On the hardware side, deploy high-precision sensors, stable data transmission networks, and efficient computing and control devices. To optimize the practical application effect, sensors should be calibrated regularly to ensure data accuracy, and data cleaning, standardization and other processing methods should be used to improve data quality. Continuously optimize model algorithms and dynamically adjust model parameters using machine learning techniques. Strengthen software and hardware collaboration, optimize sensor network layout, and enhance the parallel computing capability of computing devices. At the same time, design a simple and intuitive user interaction interface that supports multi terminal access and real-time data push.

## 7. Limitations

However, there are also certain shortcomings in the research. Firstly, in terms of methodology, the five dimensional digital twin model has a strong dependence on the quality of initial data. If sensor failures result in missing or distorted real-time rainfall and pump station water level data, the simulation accuracy of the model will decrease to below 80%. Future research can introduce multi-source data fusion algorithms to integrate meteorological stations, distributed sensors, and satellite remote sensing data, reducing the impact of single point data errors. And the dynamic scheduling algorithm does not fully consider the collaborative conflicts of multiple pump stations in emergency response under extreme weather conditions, which may result in delayed scheduling instructions. Future research could add a “multi pump station emergency collaboration module” to dynamic scheduling algorithms, resolving scheduling conflicts through a priority matrix to ensure that command response time under extreme weather conditions is ≤ 10s.

Secondly, in terms of results, the experimental validation was only based on data from a single city and did not validate the model’s universality in different climate and city size scenarios. Future research can supplement experimental data from multiple regions such as Shanghai and Xi’an, analyze the impact of climate and urban size on model parameters, and establish a parameter adaptive adjustment mechanism.

## Supporting information

S1 File Minimal data set definition. (DOCX)

S2 File Code file. (DOCX)
