# Supplementary material for: Modelling and simulation of smart city drainage system based on digital twin five-dimensional models
Source: PLoS One. 2026 Jul 9;21(7):e0352787. doi: 10.1371/journal.pone.0352787 (PMC13349131; doi:10.1371/journal.pone.0352787)
Supplement: S1 File — (DOCX) [file pone.0352787.s001.docx]

**The data in Figure 9**

| Algorithm | (a) Comparison of overflow loss between FPS algorithm and MDPIS algorithm (Scenario 1) | | | | | | | | |
| --- | --- | --- | --- | --- | --- | --- | --- | --- | --- |
|  | Scheduling Cycle | | | | | | | | |
|  | 0 | 5 | 10 | 15 | 20 | 25 | 30 | 35 | 40 |
| Unscheduled algorithm | 20 | 40 | 20 | 40 | 20 | 40 | 20 | 40 | 20 |
| FPS algorithm | / | 40 | 15 | 46 | 16 | 46 | 16 | 46 | 19 |
| MDPIS algorithm | / | / | / | 23 | 9 | 26 | 13 | 32 | 16 |
| Algorithm | (b) Comparison of overflow loss between FPS algorithm and MDPIS algorithm (Scenario 2) | | | | | | | | |
|  | Scheduling Cycle | | | | | | | | |
|  | 0 | 5 | 10 | 15 | 20 | 25 | 30 | 35 | 40 |
| Unscheduled algorithm | 20 | 25 | 30 | 35 | 40 | 35 | 30 | 25 | 20 |
| FPS algorithm | / | / | 32 | 42 | 50 | 43 | 35 | 25 | 16 |
| MDPIS algorithm | / | / | / | 22 | 29 | 29 | 26 | 18 | 14 |

**The data in Figure 10**

| Algorithm | (a) Comparison of overflow loss between FPS algorithm and MDPIS algorithm (Scenario 3) | | | | | | | | |
| --- | --- | --- | --- | --- | --- | --- | --- | --- | --- |
|  | Scheduling Cycle | | | | | | | | |
|  | 0 | 5 | 10 | 15 | 20 | 25 | 30 | 35 | 40 |
| Unscheduled algorithm | 21 | 29 | 35 | 39 | 41 | 39 | 35 | 29 | 20 |
| FPS algorithm | / | 0 | 42 | 46 | 48 | 46 | 42 | 29 | 16 |
| MDPIS algorithm | / | / | / | 23 | 27 | 33 | 30 | 23 | 14 |
| Algorithm | (b) Comparison of overflow loss between FPS algorithm and MDPIS algorithm (Scenario 4) | | | | | | | | |
|  | Scheduling Cycle | | | | | | | | |
|  | 0 | 5 | 10 | 15 | 20 | 25 | 30 | 35 | 40 |
| Unscheduled algorithm | 30 | 30 | 30 | 30 | 30 | 30 | 30 | 30 | 30 |
| FPS algorithm | / | 34 | 34 | 34 | 34 | 34 | 34 | 34 | 34 |
| MDPIS algorithm | / | / | / | 13 | 20 | 26 | 26 | 26 | 26 |

**The data in Figure 11**

| Algorithm | (a) Comparison of the number of overflow points before and after renovation | | | | (b) Comparison of average overflow time before and after renovation | | | |
| --- | --- | --- | --- | --- | --- | --- | --- | --- |
|  | Return period P(a) | | | | Return period P(a) | | | |
|  | 1 | 3 | 10 | 30 | 1 | 2 | 10 | 20 |
| Before renovation | 4 | 5 | 7 | 8 | 0.25 | 0.27 | 0.31 | 0.32 |
| After transformation | 1 | 1 | 3 | 3 | 0.06 | 0.19 | 0.23 | 0.29 |

**The data in Figure 12**

| Algorithm | (a) Comparison of the maximum average overflow time before and after renovation | | | | (b) Comparison of average total overflow volume before and after renovation | | | |
| --- | --- | --- | --- | --- | --- | --- | --- | --- |
|  | Return period P(a) | | | | Return period P(a) | | | |
|  | 1 | 2 | 10 | 20 | 1 | 2 | 10 | 20 |
| Before renovation | 0.37 | 0.46 | 0.39 | 0.37 | 29.75 | 37.39 | 44.48 | 46.80 |
| After transformation | 0.34 | 0.34 | 0.34 | 0.34 | 2.75 | 6.21 | 14.96 | 23.80 |

**The data in Figure 13**

| / | Time (h·m) | | | | | | | |
| --- | --- | --- | --- | --- | --- | --- | --- | --- |
|  | 0:21 | 0:41 | 1:01 | 1:21 | 1:41 | 2:01 | 2:21 | 2:41 |
| Return period after renovation 1a | / | 0.0652 | 0.0858 | 0.0436 | 0.0305 | 0.0174 | 0.0035 | 0.0010 |
| Return period after renovation 3a | / | 0.0982 | 0.1173 | 0.0652 | 0.0372 | 0.0223 | 0.0062 | 0.0025 |
| Return period after renovation 10a | / | 0.1309 | 0.1284 | 0.0831 | 0.0454 | 0.0305 | 0.0074 | 0.0025 |
| Return period after renovation 30a | / | 0.1495 | 0.1396 | 0.0942 | 0.0496 | 0.0355 | 0.0087 | 0.0025 |
| Return period before renovation 1a | 0.0372 | 0.2177 | 0.1309 | 0.0831 | 0.0479 | 0.0310 | 0.0074 | 0.0020 |
| Return period before renovation 3a | 0.0503 | 0.2380 | 0.1396 | 0.1004 | 0.0615 | 0.0434 | 0.0082 | 0.0025 |
| Return period before renovation 10a | 0.0640 | 0.2487 | 0.1545 | 0.1141 | 0.0818 | 0.0479 | 0.0082 | 0.0025 |
| Return period before renovation 10a | 0.0739 | 0.2636 | 0.1607 | 0.1227 | 0.0925 | 0.0595 | 0.0082 | 0.0020 |
