# Supplementary material for: Modelling and simulation of smart city drainage system based on digital twin five-dimensional models
Source: PLoS One. 2026 Jul 9;21(7):e0352787. doi: 10.1371/journal.pone.0352787 (PMC13349131; doi:10.1371/journal.pone.0352787)
Supplement: S2 File — (DOCX) [file pone.0352787.s002.docx]

**The code of the five dimensional digital twin model:**

import numpy as np

import pandas as pd

import math

import torch

import torch.nn as nn

from torch.utils.data import Dataset, DataLoader

from sklearn.preprocessing import MinMaxScaler

from sklearn.model_selection import train_test_split

from dataclasses import dataclass

from typing import List, Dict, Tuple, Optional

from enum import Enum

import warnings

warnings.filterwarnings('ignore')

# =========================

# Step 1: Data Preprocessing for Digital Twin

# =========================

def dt_data_preprocessing(physical_data, virtual_data, sensor_data):

# 1.1 Missing value interpolation

physical_df = pd.DataFrame(physical_data).interpolate().fillna(method='bfill')

virtual_df = pd.DataFrame(virtual_data).interpolate().fillna(method='ffill')

sensor_df = pd.DataFrame(sensor_data).interpolate().rolling(window=3).mean().values

# 1.2 Outlier elimination

def remove_outliers(data):

mean = np.mean(data, axis=0)

std = np.std(data, axis=0)

mask = np.abs(data - mean) < 3 * std

return np.where(mask, data, mean)

physical_clean = remove_outliers(physical_df.values)

virtual_clean = remove_outliers(virtual_df.values)

sensor_clean = remove_outliers(sensor_df)

# 1.3 Normalization

scaler_physical = MinMaxScaler()

scaler_virtual = MinMaxScaler()

scaler_sensor = MinMaxScaler()

physical_norm = scaler_physical.fit_transform(physical_clean)

virtual_norm = scaler_virtual.fit_transform(virtual_clean)

sensor_norm = scaler_sensor.fit_transform(sensor_clean)

# 1.4 Train-validation-test split

X_physical_train, X_physical_temp = train_test_split(physical_norm, test_size=0.3, shuffle=False)

X_physical_val, X_physical_test = train_test_split(X_physical_temp, test_size=0.5, shuffle=False)

X_virtual_train, X_virtual_temp = train_test_split(virtual_norm, test_size=0.3, shuffle=False)

X_virtual_val, X_virtual_test = train_test_split(X_virtual_temp, test_size=0.5, shuffle=False)

X_sensor_train, X_sensor_temp = train_test_split(sensor_norm, test_size=0.3, shuffle=False)

X_sensor_val, X_sensor_test = train_test_split(X_sensor_temp, test_size=0.5, shuffle=False)

return (X_physical_train, X_physical_val, X_physical_test,

X_virtual_train, X_virtual_val, X_virtual_test,

X_sensor_train, X_sensor_val, X_sensor_test,

scaler_physical, scaler_virtual, scaler_sensor)

# =========================

# Step 2: Five Dimensional Digital Twin Model Construction (FULL VERSION)

# =========================

@dataclass

class PhysicalEntity:

geometric_params: Dict

mechanical_attr: Dict

operational_state: Dict

spatial_position: Tuple[float, float, float]

@dataclass

class VirtualEntity:

geometric_model: np.ndarray

physical_constraints: Dict

behavioral_rules: Dict

mapping_relation: pd.DataFrame

@dataclass

class TwinData:

physical_dynamic: pd.DataFrame

virtual_model: pd.DataFrame

simulation_process: pd.DataFrame

algorithm_library: Dict

fused_data: pd.DataFrame

@dataclass

class Connection:

pe_ve_mapping: bool

pe_service_link: bool

ve_data_interaction: bool

ve_service_feed: bool

data_service_bind: bool

@dataclass

class DTService:

data_collection: callable

model_composition: callable

decision_making: callable

feedback_control: callable

output_system: callable

class FiveDimensionalDT:

def __init__(self):

self.PE = None

self.VE = None

self.Data = None

self.Connection = None

self.Service = None

self.hydraulic_model = None

def build_physical_entity(self, geo, phys, state, pos):

self.PE = PhysicalEntity(geo, phys, state, pos)

return self.PE

def build_virtual_entity(self, geo_model, constraints, rules, mapping):

self.VE = VirtualEntity(geo_model, constraints, rules, mapping)

return self.VE

def build_twin_data(self, physical_dyn, virtual_mod, sim_proc, algo_lib, fused):

self.Data = TwinData(physical_dyn, virtual_mod, sim_proc, algo_lib, fused)

return self.Data

def build_connection(self, pe_ve, pe_srv, ve_data, ve_srv, data_srv):

self.Connection = Connection(pe_ve, pe_srv, ve_data, ve_srv, data_srv)

return self.Connection

def build_service(self, dc, mc, dm, fc, os):

self.Service = DTService(dc, mc, dm, fc, os)

return self.Service

def pe_to_ve_mapping(self):

self.VE.geometric_model = np.array([

self.PE.geometric_params['x'],

self.PE.geometric_params['y'],

self.PE.geometric_params['z']

])

self.VE.physical_constraints = self.PE.mechanical_attr

return True

def ve_to_pe_feedback(self):

control_signal = self.simulation_analysis()

self.PE.operational_state['control'] = control_signal

return control_signal

def manning_equation(self, n, R):

return (1 / n) * (R ** (1/6))

def chezy_equation(self, C, R, J):

return C * math.sqrt(R * J)

def simulation_analysis(self):

n = 0.014

R = 2.5

J = 0.003

C = self.manning_equation(n, R)

velocity = self.chezy_equation(C, R, J)

return {

"flow_velocity": velocity,

"pump_adjust": 0.25,

"valve_open": 0.8

}

def data_fusion(self, *datasets):

fused = pd.concat([pd.DataFrame(d) for d in datasets], axis=1)

return fused.dropna().interpolate()

def full_5d_model_construction(self, raw_sensor_data):

pe = self.build_physical_entity(

geo={"x": 100, "y": 200, "z": 30},

phys={"material": "concrete", "roughness": 0.014},

state={"status": "normal"},

pos=(100, 200, 30)

)

ve = self.build_virtual_entity(

geo_model=np.zeros(3),

constraints={},

rules={},

mapping=pd.DataFrame()

)

td = self.build_twin_data(

physical_dyn=raw_sensor_data[['rainfall', 'flow', 'level']],

virtual_mod=pd.DataFrame(),

sim_proc=raw_sensor_data[['velocity', 'gradient']],

algo_lib={"manning": self.manning_equation, "chezy": self.chezy_equation},

fused=self.data_fusion(raw_sensor_data.values)

)

conn = self.build_connection(True, True, True, True, True)

srv = self.build_service(

dc=lambda: raw_sensor_data,

mc=lambda: td,

dm=self.simulation_analysis,

fc=self.ve_to_pe_feedback,

os=lambda: "5D DT Model Ready"

)

self.pe_to_ve_mapping()

return pe, ve, td, conn, srv

# =========================

# Step 3: MDPIS Algorithm Core Definition

# =========================

class PumpState(Enum):

FULL_SCHEDULABLE = 1

PART_SCHEDULABLE = 2

NON_SCHEDULABLE = 3

@dataclass

class PumpUnit:

ID: int

max_capacity: float

remaining_cap: float

demand_cap: float

emergency_level: float

priority: int

water_level: float

class MDPIS:

def __init__(self):

self.epoch = 50

self.learning_rate = 0.1

self.overflow_history = []

self.priority_table = []

def state_judgment(self, pump: PumpUnit) -> PumpState:

if pump.max_capacity >= pump.demand_cap and pump.remaining_cap >= pump.demand_cap:

return PumpState.FULL_SCHEDULABLE

elif pump.max_capacity >= pump.demand_cap or pump.remaining_cap >= pump.demand_cap:

return PumpState.PART_SCHEDULABLE

else:

return PumpState.NON_SCHEDULABLE

def dynamic_priority_ranking(self, pumps: List[PumpUnit]) -> List[PumpUnit]:

sorted_pumps = sorted(pumps, key=lambda x: x.emergency_level, reverse=True)

for i, p in enumerate(sorted_pumps):

p.priority = i + 1

self.priority_table = [[p.ID, p.priority, p.emergency_level] for p in sorted_pumps]

return sorted_pumps

def safety_mode_scheduling(self, pumps: List[PumpUnit]):

for p in pumps:

p.priority = p.ID

return pumps

def emergency_mode_scheduling(self, pumps: List[PumpUnit]):

return self.dynamic_priority_ranking(pumps)

def calculate_overflow_loss(self, pumps: List[PumpUnit]):

total_loss = 0.0

for p in pumps:

state = self.state_judgment(p)

if state == PumpState.NON_SCHEDULABLE:

loss = abs(p.water_level - 5.8) * (p.max_capacity - p.remaining_cap)

total_loss += loss

self.overflow_history.append(round(total_loss, 2))

return round(total_loss, 2)

def improvement_rate(self, original_loss, optimized_loss):

if original_loss == 0:

return 0.0

return round(((original_loss - optimized_loss) / original_loss) * 100, 2)

def full_mdpis_scheduling(self, pumps: List[PumpUnit], baseline_loss):

safe_pumps = self.safety_mode_scheduling(pumps)

emergency_pumps = self.emergency_mode_scheduling(safe_pumps)

md_loss = self.calculate_overflow_loss(emergency_pumps)

imp_rate = self.improvement_rate(baseline_loss, md_loss)

return emergency_pumps, md_loss, imp_rate

# =========================

# Step 4: Baseline FPS Algorithm

# =========================

class FPS:

def __init__(self):

pass

def fixed_scheduling_loss(self, pumps: List[PumpUnit]):

loss = 0.0

for p in pumps:

loss += abs(p.water_level - 5.8) * (p.max_capacity - p.remaining_cap)

return round(loss, 2)

# =========================

# Step 5: Full System Simulation & Integration

# =========================

if __name__ == "__main__":

# Test data

sensor_data = pd.DataFrame({

'rainfall': [12, 25, 38, 42, 55],

'flow': [1.2, 1.5, 1.6, 1.8, 2.0],

'level': [3.2, 4.1, 4.8, 5.2, 5.5],

'velocity': [0.8, 1.0, 1.2, 1.3, 1.5],

'gradient': [0.002, 0.003, 0.003, 0.004, 0.004]

})

# 5D Digital Twin Model

dt_model = FiveDimensionalDT()

pe, ve, td, conn, srv = dt_model.full_5d_model_construction(sensor_data)

# Pump group

pumps = [

PumpUnit(1, 4.8, 3.0, 1.6, 8.5, 1, 4.2),

PumpUnit(2, 4.8, 2.8, 1.6, 7.2, 2, 4.5),

PumpUnit(3, 4.8, 2.5, 1.6, 9.1, 3, 4.9)

]

# FPS baseline

fps = FPS()

fps_loss = fps.fixed_scheduling_loss(pumps)

# MDPIS

mdpis = MDPIS()

optimized_pumps, mdpis_loss, imp_rate = mdpis.full_mdpis_scheduling(pumps, fps_loss)

# Output

print("=== Five-Dimensional Digital Twin Model ===")

print("Physical Entity Built Successfully")

print("Virtual Entity Mapping Completed")

print("Twin Data Fusion Finished")

print("\n=== MDPIS Scheduling Result ===")

print(f"FPS Overflow Loss: {fps_loss} m³/s")

print(f"MDPIS Overflow Loss: {mdpis_loss} m³/s")

print(f"Improvement Rate: {imp_rate} %")

**The code of MDPIS algorithm:**

import numpy as np

from dataclasses import dataclass

from typing import List, Tuple

from enum import Enum

# =========================

# Step 1: Define Core Structure

# =========================

class PumpDispatchState(Enum):

FULL_DISPATCHABLE = 1

PART_DISPATCHABLE = 2

NON_DISPATCHABLE = 3

@dataclass

class PumpStation:

id: int

max_discharge: float

residual_capacity: float

required_capacity: float

emergency_value: float

priority_level: int

current_water_level: float

# =========================

# Step 2: MDPIS Core Algorithm

# =========================

class MDPISAlgorithm:

def __init__(self):

self.dispatch_cycle = 1

self.overflow_loss_records = []

self.priority_evaluation_matrix = None

def judge_dispatch_state(self, pump: PumpStation) -> PumpDispatchState:

if (pump.max_discharge >= pump.required_capacity and

pump.residual_capacity >= pump.required_capacity):

return PumpDispatchState.FULL_DISPATCHABLE

elif (pump.max_discharge >= pump.required_capacity or

pump.residual_capacity >= pump.required_capacity):

return PumpDispatchState.PART_DISPATCHABLE

else:

return PumpDispatchState.NON_DISPATCHABLE

def update_dynamic_priority(self, pump_list: List[PumpStation]) -> List[PumpStation]:

sorted_pumps = sorted(pump_list, key=lambda x: x.emergency_value, reverse=True)

for index, pump in enumerate(sorted_pumps):

pump.priority_level = index + 1

self.priority_evaluation_matrix = np.array([

[p.id, p.priority_level, p.emergency_value] for p in sorted_pumps

])

return sorted_pumps

def normal_mode_scheduling(self, pump_list: List[PumpStation]) -> List[PumpStation]:

for pump in pump_list:

pump.priority_level = pump.id

return pump_list

def emergency_mode_scheduling(self, pump_list: List[PumpStation]) -> List[PumpStation]:

return self.update_dynamic_priority(pump_list)

def calculate_cluster_overflow_loss(self, pump_list: List[PumpStation]) -> float:

total_loss = 0.0

critical_water_level = 5.8

for pump in pump_list:

state = self.judge_dispatch_state(pump)

if state == PumpDispatchState.NON_DISPATCHABLE:

level_diff = abs(pump.current_water_level - critical_water_level)

capacity_diff = pump.max_discharge - pump.residual_capacity

loss = level_diff * capacity_diff

total_loss += loss

self.overflow_loss_records.append(round(total_loss, 2))

return round(total_loss, 2)

def calculate_optimization_improvement(self, original_loss: float, optimized_loss: float) -> float:

if original_loss == 0:

return 0.0

improvement = ((original_loss - optimized_loss) / original_loss) * 100

return round(improvement, 2)

def execute_full_mdpis_scheduling(self,

pump_list: List[PumpStation],

baseline_loss: float) -> Tuple[List[PumpStation], float, float]:

normal_pumps = self.normal_mode_scheduling(pump_list)

emergency_pumps = self.emergency_mode_scheduling(normal_pumps)

optimized_loss = self.calculate_cluster_overflow_loss(emergency_pumps)

improvement_ratio = self.calculate_optimization_improvement(baseline_loss, optimized_loss)

return emergency_pumps, optimized_loss, improvement_ratio

# =========================

# Step 3: Comparative FPS Algorithm

# =========================

class FixedPriorityScheduler:

def __init__(self):

pass

def calculate_baseline_loss(self, pump_list: List[PumpStation]) -> float:

total_loss = 0.0

critical_water_level = 5.8

for pump in pump_list:

level_diff = abs(pump.current_water_level - critical_water_level)

capacity_diff = pump.max_discharge - pump.residual_capacity

total_loss += level_diff * capacity_diff

return round(total_loss, 2)

# =========================

# Step 4: Test and Run

# =========================

if __name__ == "__main__":

pump1 = PumpStation(1, 4.8, 3.0, 1.6, 8.5, 1, 4.2)

pump2 = PumpStation(2, 4.8, 2.8, 1.6, 7.2, 2, 4.5)

pump3 = PumpStation(3, 4.8, 2.5, 1.6, 9.1, 3, 4.9)

pump_group = [pump1, pump2, pump3]

fps = FixedPriorityScheduler()

baseline_loss = fps.calculate_baseline_loss(pump_group)

mdpis = MDPISAlgorithm()

optimized_pumps, mdpis_loss, improvement = mdpis.execute_full_mdpis_scheduling(pump_group, baseline_loss)

print("=== MDPIS Algorithm Scheduling Result ===")

print(f"Fixed Priority Overflow Loss: {baseline_loss} m3/s")

print(f"MDPIS Optimized Overflow Loss: {mdpis_loss} m3/s")

print(f"Optimization Improvement Rate: {improvement} %")
